# The Potential of T Cell Factor 1 in Sustaining CD8^+^ T Lymphocyte-Directed Anti-Tumor Immunity

**DOI:** 10.3390/cancers13030515

**Published:** 2021-01-29

**Authors:** Sungmin Jung, Jea-Hyun Baek

**Affiliations:** School of Life Science, Handong Global University, Pohang, Gyeongbuk 37554, Korea; 21700656@handong.edu

**Keywords:** CD8, T lymphocyte, exhaustion, reinvigoration, T cell factor 1, immune checkpoint blockade, anti-tumor immunity

## Abstract

**Simple Summary:**

The transcription factor T cell factor 1 (TCF1), encoded by the *TCF7* gene, is a key regulator of T-cell fate, which is known to promote T cell proliferation and establish T cell stemness. Importantly, increasing evidence has demonstrated that TCF1 is a critical determinant of the success of anti-tumor immunotherapy, implicating that TCF1 is a promising biomarker and therapeutic target in cancer. In recent years, new findings have emerged to provide a clearer view of TCF1 and its role in T cell biology. In this review, we aim to provide a comprehensive outline of the most recent literature on the role of TCF1 in T cell development and to discuss the potential of TCF1 in sustaining CD8^+^ T lymphocyte-directed anti-tumor immunity.

**Abstract:**

T cell factor 1 (TCF1) is a transcription factor that has been highlighted to play a critical role in the promotion of T cell proliferation and maintenance of cell stemness in the embryonic and CD8^+^ T cell populations. The regulatory nature of TCF1 in CD8^+^ T cells is of great significance, especially within the context of T cell exhaustion, which is linked to the tumor and viral escape in pathological contexts. Indeed, inhibitory signals, such as programmed cell death 1 (PD-1) and cytotoxic-T-lymphocyte-associated protein 4 (CTLA-4), expressed on exhausted T lymphocytes (T_EX_), have become major therapeutic targets in immune checkpoint blockade (ICB) therapy. The significance of TCF1 in the sustenance of CTL-mediated immunity against pathogens and tumors, as well as its recently observed necessity for an effective anti-tumor immune response in ICB therapy, presents TCF1 as a potentially significant biomarker and/or therapeutic target for overcoming CD8^+^ T cell exhaustion and resistance to ICB therapy. In this review, we aim to outline the recent findings on the role of TCF1 in T cell development and discuss its implications in anti-tumor immunity.

## 1. Introduction

In recent years, studies into the regulatory effects of the transcription factor T cell factor 1 (TCF1) have become a rising trend in the CD8^+^ T cell differentiation and exhaustion research area. Exhaustion of CD8^+^ T cells during chronic viral infections and in tumor microenvironments (TME) has been highlighted as a major contributing factor that severely decreases a patient’s likelihood of survival. Despite decades of research into CD8^+^ T cells and their dysfunction, T cell exhaustion has remained yet to be fully comprehended or resolved.

To combat T cell exhaustion, immune checkpoint blockade (ICB) has been developed as a strategy of preventing the ligation of inhibitory receptors on CD8^+^ T cells using monoclonal antibody technology. While ICB has shown remarkable success in improving the survival of cancer patients, clinical studies have reported that about 50% of patients present with ICB resistance, whereas an additional average of about 20% of patients develop hyper-progression in response to ICB treatment [1,2]. Previous studies have delved into various potential strategies for delaying or preventing T cell exhaustion, including anti-vascular endothelial growth factor (VEGF) therapies and the transfer of engineered T cells expressing checkpoint inhibitors. On the other hand, new emerging studies appear to hint towards a new potential approach that utilizes cell stemness for combating T cell exhaustion while promoting effector functionality [3,4,5]. In line with this, studies have found the subset of TCF1^+^CD8^+^ T cells to be essential in mounting an effective immune response in viral infections and cancer [6,7,8]. In addition, TCF1 has been recently implicated in the regulation and determination of Foxp3^+^ regulatory T cells (Tregs) [9].

Exhausted T cells (T_EX_) present reduced proliferative capacities and dysfunctional effector responses along with cell surface expression of inhibitory receptors such as programmed cell death 1 (PD-1), cytotoxic T lymphocyte-associated protein 4 (CTLA-4), Tim3, Lag3, and T cell immunoreceptor with Ig and ITIM domain (TIGIT) [10]. Currently, there is a lively debate about the mechanism of T cell exhaustion. While some researchers perceive T cell exhaustion only as cellular dysfunction, the others view it mainly as a functional mechanism for host protection. Interestingly, TCF1-expressing CD8^+^ T cells sustain effector responses while simultaneously displaying a T_EX_ phenotype in chronic settings [8,11]. A recent study into the PD-1^+^CD8^+^ T cells in acute and convalescent COVID-19 patients found such T cells to be functional, despite the presence of PD-1 [12].

The origin and development of T_EX_ cells remain yet to be fully explored with regards to their importance for better addressing CD8^+^ T cell exhaustion and the shortcomings of ICB therapy. The cell stemness that is associated with TCF1 expression may be used for developing a novel approach to resolve CD8^+^ T cell resistance to ICB therapy in cancer patients and potentially fine-tune our current understanding of T cell exhaustion and differentiation.

## 2. T Cell Factor 1

TCF1 (encoded by *TCF7* gene) belongs to a larger T cell factor/lymphocyte enhancer factor (TCF/LEF) family of transcription factors. Vertebrates possess four TCF/LEF genes, consisting of TCF1, LEF1, TCF3, and TCF4. The TCF/LEF family members are not transcriptional factors under the most conventional of definitions, as they do not initiate DNA transcription independently. TCF/LEF proteins have DNA binding capabilities through the Wnt-response element (WRE) conserved in their high-mobility group (HMG) domain [13]. Of note, initial studies identified these as factors forming a nuclear-binding complex with β-catenin and inducing the expression of its target genes [14,15].

As direct downstream signaling mediators of the Wnt/β-catenin pathway, the TCF/LEF transcription factors have been greatly implicated in the regulation of T cell stemness and differentiation. It has been shown that TCF1 expression levels are vastly upregulated in naïve and memory CD8^+^ T cells in contrast to effector and terminally differentiated CD8^+^ T cells [16]. Indeed, downregulation of TCF1 was found to be essential for CD8^+^ T cell differentiation into short-lived effector cells (SLECs) and terminally differentiated T cells in both acute and chronic infections, respectively. Furthermore, studies have demonstrated that TCF-1 is important for the development of T_EX_ stem cells that replenish terminally differentiated T_EX_ cells following viral infection and in response to tumor formation [17]. Studies into the mechanisms of TCF1 downregulation have revealed that inflammatory stimulus, such as IL-12, decreases TCF1 and IL-2 levels in CD8^+^ T cells while enhancing expression of interferon (IFN)-γ and granzyme B [18]. The downregulation of TCF1 induces a SLEC-like phenotype (CD127^−^KLRG1^+^), whereas the upregulation of TCF1 transforms CD8^+^ T cells to a memory precursor effector cell (MPEC)-like phenotype (KLRG1^−^CD127^+^) [18]. Moreover, TCF1 regulation is found to be essential for the formation of central memory T (T_CM_) cells. In *Tcf7*^−/−^ mice, the memory T cell population is noted to be predominantly CD127^+^CD62L^−^, which correlates to an effector memory (T_EM_) phenotype, and T_CM_ cells are decreased by tenfold [18].

In CD8^+^ T_EX_ cells, the levels of TCF1 are substantially reduced. DNA methylation analysis of in vitro generated CD8^+^ T_EX_ cells in a lymphocytic choriomeningitis virus (LCMV) infection-based model of T cell exhaustion has revealed that the promoter of the *Tcf7* gene is hypermethylated, likely leading to the downregulation of TCF1 expression [19]. Similarly, previous studies have also suggested that TCF1 expression may be regulated through the control of chromatin accessibility [20,21].

Since the 1990s, studies have suggested that TCF1 and LEF1 may have redundant functions due to their remarkable structural similarities, corroborated by various experiments using TCF1 and LEF1 mutants [22,23,24,25]. Later, it was found that specific TCF1 isoforms exert unique functions, which cannot be compensated by LEF1 isoforms. In natural killer (NK) cells, many TCF1 functions can also be executed by LEF1, whereas a specific TCF1 isoform associated with the expression of NK receptor Ly49 cannot be functionally replaced by LEF1 isoforms [26]. A recent study has shed light on the divergent roles of TCF1 and LEF1 in embryonic stem cells. In embryonic stem cells, TCF1 appears to be essential for cell stemness and the expression of self-renewal genes, such as Oct4, Sox2, and Nanog, while LEF1 is more associated with the initiation of cell differentiation through its capacity for replacing TCF3 repressors at the promoters of cell differentiation-related genes [27]. Interestingly, the inhibitory role of TCF1 on cell differentiation seems to be cell type-dependent: while TCF1 completely inhibits the differentiation of neural precursors, it only slows down and delays the differentiation of cardiomyocytes, as evidenced by changes in the expression of the stemness marker Nanog [26]. Similarly, the magnitude of TCF1-mediated differentiation inhibition may vary amongst the various T cell subsets. Thus, it may be important to view TCF1 in a subset-specific manner.

In addition to the involvement of TCF1 at the embryonic stages of life, TCF1 holds significance in human aging. Both TCF1 and LEF1 are downregulated in immune cells in older individuals due to reduced chromatin accessibility at their respective gene loci [28]. Hypermethylation of the *Tcf7* gene is another suggested hindering factor towards TCF1 expression, especially given the accelerated differentiation of naïve and memory CD8^+^ T cells amongst the older populous [29,30]. Given that over 70% of cancers occur in individuals over the age of 50, the gradual loss of TCF1 expression amongst the host immune cells may cause a reduction in the ability of the host to properly respond against and clear new neoplasms [31]. Thus, TCF1 may be a major determining factor to the development of cancer and pathogen susceptibility of older individuals [32].

## 3. CD8^+^ T Lymphocytes: Heterogeneity & Differentiation

### 3.1. CD8^+^ Effector T (T_EFF_) Cells

CD8^+^ T lymphocytes have long been recognized as key mediators of cellular immunity in response to foreign pathogens and tumors. Following the recognition of an immunogenic antigenic challenge, activated CD8^+^ T cells undergo a series of clonal expansions to inflate the pool of antigen-specific clones, which, in turn, give rise to effector and memory T lymphocytes. T_EFF_ cells are further subdivided into two distinct subgroups according to their phenotype: (1) SLECs (KLRG1^+^CD127^−^), which are considered to possess the most cytotoxic effector function and harbor the highest levels of granzyme B; (2) MPECs (KLRG1^−^CD127^+^), which are characterized by higher survivability and greater potential to give rise to long-lived memory T cells [33,34].

Given such phenotypic and functional heterogeneity, two different models of memory lymphocyte differentiation have been proposed: a linear differentiation where memory cells are derived from cells that have transitioned through an effector phase and a divergent differentiation, which delineates memory and terminal effector lymphocytes as two distinct subsets [35]. Contrary to differentiation studies in plasma and memory B cells, studies appear to be evident for a linear model of memory CD8^+^ T cell differentiation. Specifically, several studies have linked the necessity of antigen exposure and effector cell proliferation for the development of memory T cells [36,37,38,39,40]. An in vivo study on the cytotoxic potential of MPECs and SLECs presented that KLRG1^hi^ and ^-int^ cells possess near equivalent capacities for CD107a/b-expressing degranulation and a key determining factor for MPECs, which preferentially localize to lymph nodes, and peripherally localized SLEC differentiation appears to be the differing levels of antigenic exposure at their respective tissue locales [41]. As such, production of memory CD8^+^ T cells is viewed to originate from MPECs, which in turn are a subset of early effector cells that have received lower amounts of antigen stimulation as opposed to peripheral SLECs.

Before highlighting the significance of antigen recognition, much attention was also given to the influence of pro- and anti-inflammatory cytokines as well as chemokines on SLEC and MPEC differentiation. Particularly, the inflammatory cytokines IL-2, IL-15, and type I IFN have been implicated to drive CD8^+^ T_EFF_ cells towards terminal differentiation [42,43,44]. On the contrary, TGF-β has been found to suppress SLEC populations by inducing apoptotic cell death [43]. A lack of IL-2 has also been noted to drastically reduce SLEC numbers. Interestingly, MPECs were found to be able to respond and proliferate to secondary antigenic exposures despite an absence of IL-2 [42]. Importantly, inflammatory cytokines appear to be capable of independently inducing a terminally differentiated state in CD8^+^ T cells. As demonstrated by Kurachi et al., antigen-primed CXCR3^−/−^ CD8^+^ T cells in the spleen failed to migrate to marginal zones and consequently received minimal stimuli from inflammatory cytokines [45].

Thus, cytokines seem to regulate differentiation in an inflammatory context while antigenic exposure and costimulatory factors can more generally drive CD8^+^ T lymphocytes into an effector state, from which they will either become memory precursors or terminally differentiated.

### 3.2. CD8^+^ Memory T Cells

Apart from effectively responding to primary antigen exposure, the development and maintenance of immunological memory are two of the primary enabling factors for a rapid response of the adaptive immune system. Studies into memory T cell development have revealed several distinct subsets differing in both phenotype and recall responses. Two of the most well-known memory T cell subsets are CD8^+^ T_CM_ and T_EM_ cells. Recognized as the memory subset with the greatest capacity for expansive proliferation in response to memory recall stimulus, T_CM_ cells are defined as CD45RA^−^, CD45RO^+^, CCR7^+^CD44^+^, CD62L^+^, CD27^+^, CXCR3^+^, CD43^−^, KLRG1^−^, and CD127^+^; some defining markers of T_EM_ cells are CD45RA^−^, CD45RO^+^, CCR7^−^, CD44^+^, CD62L^−^, CD27^+^, CXCR3^+^, CD43^+^, KLRG1^−^, and CD127^−^ (Table 1) [46,47,48,49]. T_EM_ cells that localize to peripheral sites have the greatest cytotoxic effector responses to antigen challenge but also a reduced proliferative capacity [49,50]. The difference in the presence and absence of CCR7 and CD62L in T_CM_ and T_EM_ cells, respectively, plays a key role in distinguishing between memory cells that act as peripheral sentinels and those that home to secondary lymphatic organs (SLOs) [51].

## 4. CD8^+^ T Lymphocyte Responses in Anti-Tumor Immunity

Within the context of a CD8^+^ T cell-mediated immune response against tumors, both the effector and memory CD8^+^ subpopulations have been implicated as essential for proper clearance of tumors.

### 4.1. Effector CD8^+^ T Cell-Mediated Immunity against Tumors

CD8^+^ T_EFF_ cells (cytotoxic T lymphocytes; CTLs) are key players in anti-tumor immunity, as they can directly eliminate tumor cells [57]. Naïve CD8^+^ T cells reside in the secondary lymphatic organs, until they are primed by antigen-presenting cells that present tumor antigens (either intracellular or cross-presented) via class I major histocompatibility complex (MHC). Following antigen priming and clonal expansion, naïve CD8^+^ T cells become CD8^+^ T_EFF_ cells and lose their CCR7 receptors to migrate into the peripheral tumor tissue.

In a setting of acute exposure to immunogenic antigens, CTLs have been characterized as possessing various mechanisms by which their cytotoxic functions can be activated to target abnormal cells (i.e., T cell receptor (TCR)/MHC and Toll-like receptors). Upon activation, the primary form of cytotoxicity mediated by CD8^+^ T cells is through degranulation and the exposure of the target cell to granzyme B. Following the elimination of tumor cells, the magnitude of antigen-specific CD8^+^ T_EFF_ cells decreases along with an influx of key regulatory stimuli such as Tregs and TGF-β cytokines that mediate restoration of tissue homeostasis. Given the heterogeneity of the T_EFF_ cell population, it is generally believed that a majority of the SLEC population are eliminated in the resolution phase while the MPEC population persist by giving rise to memory CD8^+^ T cells [58].

### 4.2. Memory CD8^+^ T Cell-Mediated Immunity against Tumors

Memory T cells have been suggested to present a much more competent immunoreactive efficacy in contrast to naïve T cells. Particularly, several studies into tumor-infiltrating lymphocytes (TIL) have highlighted the necessity of a healthy T_CM_ population for an active immune response under situations of chronic antigen stimulation, such as infections with LCMV [46]. The importance of T_CM_ cells may extend beyond viral infections to include tumors as well.

Such a necessity may be due in part to the ability of TCRs on memory T cells to efficiently transduce activating signals, even in the absence of co-stimulation, with an antigen exposure dosage that is 100-fold lower than the dosage required by TCR activation of naïve T cells without co-stimulation [59]. However, given the heterogeneity within memory CD8^+^ T cells, this generally lower dosage requirement may not apply across all memory subtypes. Of note, T_CM_ lymphocytes were observed to possess lower levels of TCR expression and greater amounts of protein tyrosine phosphatases in vivo, resulting in lower Zap70 activation and c-Myc expression, subsequently demanding a greater amount of antigen exposure [60]. This need for greater antigen stimulation may be met by the T_CM_ cell expression of CCR7 and the consequent interaction with dendritic cells (DCs) in the lymph nodes.

The role of T_CM_ cells in anti-tumor immunity may further be highlighted by the observation that CCR7^+^ T_CM_ cells were found to have near equivalent or even greater levels of cytokine production and cytolytic activity as that of CCR7^−^ T_EM_ cells [61,62]. Additionally, T_CM_ cells were characterized as possessing a tendency for prolonged persistence as well as antigen-stimulated proliferation and production of IL-2 [46]. Tissue-resident memory CD8^+^ T (T_RM_) cells play central roles against solid tumors. In a recent study, it was shown that tumor antigen-specific activation of T_RM_ cells induce dermal DCs to mature and migrate into the draining lymph node, ultimately triggering the spread of CD8^+^ T_EFF_ responses [63].

## 5. Exhaustion of CD8^+^ T Lymphocytes

Unfortunately, CD8^+^ T cells have been reported to be dysfunctional within a chronic infection context. Such dysfunctional CD8^+^ T cells, termed as being in an “exhausted” state, were first described over 20 years ago in in vivo chronic LCMV infections of mice [64]. CD8^+^ T_EX_ cells were found to lack proper effector function and thereby allowed viral pathogens to persist within a host [65]. Exhaustion of CD8^+^ T cells has been found in other various types of pathogenic infections and human diseases associated with a chronic presence of antigens.

### 5.1. Mechanisms of CD8^+^ T Cell Exhaustion

Under conditions of chronic antigen exposure, such as those induced by cancer, several studies have found that exhaustion leads to the loss of certain effector functions before inducing dysfunction of others [10,66,67]. Although exhaustion may vary depending on the infection or disease, CD8^+^ T cells were found to lose their proliferative capacity, as marked by a reduction in IL-2 production, and cytotoxic capabilities [67]. This could be followed by a loss in the ability to produce tumor necrosis factor among other functions. Under several viral loads, studies have found that CD8^+^ T cells can partially, or even completely, lose their ability to produce IFN-γ or carry out cytotoxic functions via degranulation [68]. Finally, at terminal exhaustion, antigen-specific T cells underwent apoptotic deletion [67,69].

Exhaustion has further been reported to alter the formation of memory CD8^+^ T cells. Considered as one the major hallmarks of memory CD8^+^ T cells, the potential for persistence and self-renewal through IL-7 and IL-15 is absent in CD8^+^ T_EX_ cells. Specifically, reduced expression of CD122, the β-chain of the IL-2 and IL15 receptors, and CD127, the ⍺-chain of the IL-7 receptor, correspond to the poor response of CD8^+^ T cells to IL-15 and IL-7 [70]. Thus, CD8^+^ T_EX_ cells were found to be dependent on antigen-TCR signaling for long-term persistence [71]. The low expression levels of CD127 found in in vivo mice studies have also been confirmed in human HIV patients, and thus have been suggested as hinting at the possibility for human CD8^+^ T cells to also be reliant on antigen-TCR stimulation for cell survival [70]. Further evidence of a lack of memory formation comes from studies that revealed low numbers of memory CD8^+^ T cells amongst CD8^+^ T_EX_ cells following antigen clearance [72]. Such a finding has been suggested as implicating that a commitment of CD8^+^ T cells to exhaustion prevents those cells from engaging in normal differentiation into memory cells, even in the absence of antigens [73].

### 5.2. Inhibitory Immune Checkpoints

As one of the earliest inhibition receptors identified on T cells, the function of CTLA-4 has been determined as being competitive with CD28 for binding to B7 molecules, found on the surface of APCs. By preventing the ligation between CD28 and B7, T cells are unable to receive the co-stimulatory signals necessary for proper T cell activation. In this manner, CTLA-4 interferes with the priming stage of T cells. PD-1, on the other hand, confers dysfunction of effector-related responses to CD8^+^ T cells. PD-1 is expressed on the surface of activated T cells, similar to T cells; however, the binding of PD-1 to its ligand PD-L1 can cause already activated CD8^+^ T cells to cease effector functions. Thus, CTLA-4 is deemed to be more correlated to the priming stage of T cells whereas PD-1 is more associated with the dysfunction of an already primed cell [74].

### 5.3. CD8^+^ T_EX_ Cells in the TME

The dysfunctional execution of anti-tumor immunity by CD8^+^ T_EX_ cells is further hampered by TME. In addition to the presentation of immune checkpoint ligands by the cancer cells, tumors recruit various types of immune cells, such as macrophages and myeloid cells, capable of discouraging the infiltration of CD8^+^ T cells (TILs) into the TME. Tumor-associated macrophages (TAMs), which constitute the major leukocyte population within the TME, have been found to support the survival of tumors through tissue repair and the secretion of anti-inflammatory cytokines, including TGF-β [75]. The tissue repair triggered by TAMs has been suggested as creating an extracellular matrix that makes accessing the TME more challenging for CD8^+^ T cells [76]. Myeloid-derived suppressor cells (MDSCs) are a second class of immune cells of myeloid origin found to promote tumor survival. Though the exact lineage of MDSCs is still unclear, studies have found a mix of both monocytic and polymorphonuclear characteristics. In particular, studies using mouse-derived MDSCs have reported that monocytic MDSCs seem to present an immunosuppressive function in an antigen-independent manner whereas polymorphonuclear-like MDSCs inhibit the effector functions of antigen-specific CD8^+^ T cells [77,78]. TAMs and MDSCs have both been specifically linked to the production of reactive oxygen species (ROS), capable of antagonizing both the expression of CD3ζ and its association with TCR [79,80]. Finally, MDSCs have also been found to engage immune checkpoint receptors by presenting PD-L1, PD-L2, and B7, targets of PD-1 and CTLA-4 [81,82,83].

## 6. ICB as a Method of T Cell Reinvigoration

Since the discovery of immune checkpoints and monoclonal antibody-mediated targeting of specific cellular receptors, ICB has emerged as one of the most successful forms of immunotherapy for cancer treatment, showing efficacy in even metastatic and conventional treatment-resistant forms of cancer. ICB has predominantly focused on targeting the inhibitory receptors CTLA-4 and PD-1 as well as PD-L1 [84].

As of now, only four types of ICB monoclonal antibodies have received FDA approval for use in human treatment. The first to receive such approval was ipilimumab, a target of CTLA-4, for treatment of melanoma, nivolumab, and pembrolizumab, targets of PD-1, obtained their first approval in 2014 for the treatment of melanoma. Atezolizumab, a target for PD-L1, received its first approval in 2016 for use in the treatment of urothelial carcinoma. In the following years, these monoclonal antibodies have been approved as treatment options for several other types of cancers [85].

Treatments using ICB have shown great results in providing cancer patients with an alternative method of treatment. Numerous cases of tumor regression and successful CD8^+^ T cell reinvigoration were noted by clinical studies to significantly increase patient survival rates [86].

### 6.1. Resistance to ICB Therapy

Despite its remarkable success, the rate at which many patients develop resistance to ICB therapy has been noted as a considerable concern. When taking into consideration the frequency of ICB resistance and hyper-progression, the rate of failure for ICB treatment amounts to about 60~80% of treated patients [1,2]. Studies into the resistance mechanisms by cancer cells have revealed cellular alterations that cause CD8^+^ T cells to fail in eliminating tumors include: (1) upregulation of inhibitory receptors/ligands, (2) loss of neoantigens, (3) disruption of MHC antigen presentation due to a loss of β2 microglobulin, (4) activation of oncogenic pathways, (5) mutations in IFN-γ signaling, and (6) increased recruit of suppressor immune cells (i.e., Tregs, tumor-associated macrophages (TAMs), and MDSCs) [87].

### 6.2. The Role of TCF1 in ICB and CD8^+^ T Cell Reinvigoration

**Figure 1:**
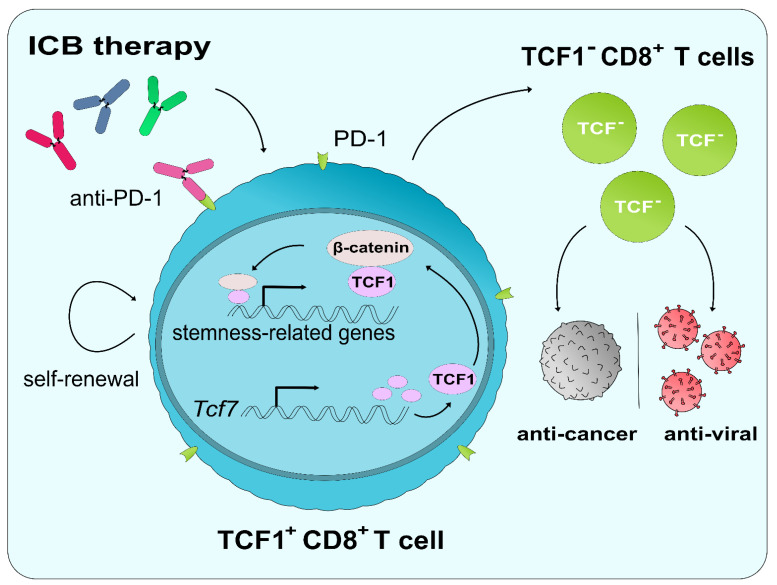
A suggested model of the impact of TCF1 expression in the regulation of T cell differentiation under chronic conditions of infection and cancer. TCF1^+^CD8^+^ T cells give rise to TCF1^−^CD8^+^ T_EFF_ cells, which carry out anti-cancer and anti-viral immune responses.

Studies into CD8^+^ T cell differentiation in response to pathogenic stimulation have started to highlight the role of TCF1 in providing a pool of stem-like precursors that can give rise to TCF1^−^CD8^+^ T_EFF_ cells and provide a robust immune response (Figure 1). The significance of TCF1 within the context of ICB has just started to come to light and may offer a strategy to overcome ICB resistance. As one of the first major findings, Siddiqui et al. reported in 2019 that CD8^+^ T cells expressing TCF1 and PD-1 were responsible for mediating anti-tumor immunity upon the administration of ICB therapy [8]. An absence of TCF1 expression was noted as producing poor responses against ICB therapy. Despite the presence of exhaustion markers, such as PD-1, TCF1^+^ cells were determined to retain stem-like properties, including having the ability for exponential expansion, self-renewal, differentiation into TCF1^−^ tumor-infiltrating lymphocytes within the TME (Figure 1). These results were also confirmed by another study published in 2019 highlighting stem-like TCF1^+^PD-1^+^ T cells as providing a pool of highly proliferative cells that give rise to antigen-specific transitory T_EFF_ cells that respond to chronic viral infections [88]. Furthermore, PD-1 blockade was found to expand the pool size of these stem-like TCF1^+^PD-1^+^ T cells [88]. However, PD-1^+^ cells were not the only cell type to receive influence from TCF1. PD-1^−^CD8^+^ T MPECs were also found to be dependent on the expression of TCF1, and the conditional deletion of *Tcf7* in MPECs compromised their ability to respond to ICB therapy [7,89]. Interestingly enough; however, observations in melanoma patients reported that only the expression of both *TCF7* and *PDCD1* in CD8^+^ T cells correlated with improved patient survival rates [8]. When taken in conjunction with ICB resistance, this could be seen as a result of depleting the TCF1^+^ pool among CD8^+^ T cells. Thus, studies into alterations of the TCF1^+^ population among CD8^+^ T cells following ICB treatment in resistant patients and methods for replenishing the TCF1^+^CD8^+^ T cells may be necessary.

The role of antigen exposure and cytokines in T cell development and differentiation is relatively well-known. In recent years, studies into the role of TCF1 in its regulation of T cell differentiation have revealed the essential nature of TCF1 for the development of memory CD8^+^ T cells [90,91]. TCF1 has also been implicated as being essential for the persistence of memory CD8^+^ T cells [91]. Upon activation through the Wnt/β-catenin pathway, TCF1 as a transcription factor belonging to the TCF family can bind with the β-catenin following its translocation into the cell nucleus. The binding of β-catenin with TCF1 allows for the transient transcription of genes related to the maintenance of cell stemness, particularly Nanog [27].

However, TCF1 may be disadvantageous for the generation of T_RM_ cells. TCF1 was found to cause the downregulation of CD103 expression in vivo, a marker necessary for tissue residence [92]. Furthermore, a deficiency in TGF-β was found to cause the loss of CD103 and the restoration of TCF1 expression in T_RM_ cell precursors [92]. Such downregulation of CD103 mediated by TCF1 may allow for the systemic circulation of CD8^+^ T_RM_ cells and confer to these T_RM_ cells functions similar to those of CD8^+^ T_CM_ cells.

The potential significance of such findings linking TCF1 with memory CD8^+^ T cell formation and cell stemness comes especially into perspective when simultaneously considering the results of a previous induced pluripotent stem cell (iPSC)-based CD8^+^ T cell study. In 2013, a study introduced a novel methodology for generating iPSCs from antigen-specific CD8^+^ T_EX_ cells and redifferentiating those iPSCs back into CD8^+^ T cells [93]. Re-differentiated CD8^+^ T cells were found to have conserved their antigen specificity for their original human immunodeficiency virus (HIV) antigen, Nef-138-8 WT. More importantly, through induced un- and redifferentiation, CD8^+^ T_EX_ cells presented with enhanced proliferative capabilities due to elongated telomeres, generating a clonal expansion size from 100- to 1000-fold, as well as producing significant amounts of IFN-γ and cytotoxic 107a-expressing degranulation. These rejuvenated CD8^+^ T cells were characterized as having T_CM_ cell-like features, including the expression of CCR7, CD27, and CD28. iPSC-rejuvenated CD8^+^ T cells retained T_CM_ markers even after 100- and 1000-fold clonal expansions. Indeed, such findings of memory-like CD8^+^ T cells mediating enhanced anti-tumor immunity have been found by Siddiqui et al. as well. TCF1^+^PD-1^+^CD8^+^ T cells were found to express gene markers similar to that of MPEC and T_CM_ cells, including CD62L, CCR7, Id3, and Bcl6 [8].

Other studies have also found that memory-like TCF1^+^CD8^+^ T cells were responsible for maintaining effective T cell responses against pathogens. In particular, while found to be not as essential for initial T cell expansion, *Tcf7*^−/−^ mice presented with lower frequencies of antigen-specific CD8^+^ T cells over extended periods of observation and failed to adequately control viral titers [11]. Similar results were confirmed by a 2020 study in murine cytomegalovirus chronic infections [6]. The highly proliferative TCF1^+^CD8^+^ T cells were found to be responsible for maintaining the inflationary pool of TCF1^−^CD8^+^ T_EFF_ cells. Thus, future findings into methods for sustaining, or even inducing, TCF1 expression in CD8^+^ T cells may offer a mechanism for prolonged tumor control via ICB therapy. Interestingly, the pool of TCF1^+^CD8^+^ T cells was also reported to present a greater variety in TCR repertoire in comparison to their TCF1^−^ counterparts [6]. Within the context of the TME, this increased TCR repertoire diversity may present CD8^+^ TILs a greater potential for detecting and responding against neoantigens.

Given the importance of the presence of TCF1^+^ CD8^+^ T cells for a proper response to viral infections and ICB therapy, the levels of TCF1^+^ T cells may also be an important biomarker for determining the possible efficacy of ICB therapy in patients. Of note, the method through which a therapy is administered to a patient may be a critical factor for determining the amount of TCF1^+^CD8^+^ T cells generated in response to the therapy. As noted by Baharom et al., intravenous administration of a nanoparticle vaccine produced a significantly greater amount of stem-like TCF1^+^PD-1^+^CD8^+^ T cells in stark contrast with the administration of the vaccine via subcutaneous injection [94]. These TCF1^+^PD-1^+^CD8^+^ T cells were characterized showing a similar phenotype with stem-like T_EX_ cell progenitors, expressing eomesodermin and CXCR3.

A more radical line of thought linking the expression of TCF1 with stem-cell memory T cells (T_SCM_) and T cell reinvigoration may also be plausible. Though not as widely regarded as T_CM_ and T_EM_ cells, T_SCM_ cells, defined as expressing CD62L, CCR7, CD45RA, CD45RO, IL-7Rα, CD95, have also been correlated to positive persistence, potentially having an ability for self-renewal while suppressing and eliminating tumor cells (Table 1) [95]. Previous studies have associated T_SCM_ cells with the Wnt/β-catenin pathway as glycogen synthase kinase-3β (GSK-3β) inhibitors elevated T_SCM_ cell numbers [52,95]. Relative to T_CM_ and T_EM_ cells, T_SCM_ cells are deemed to have greater anti-tumor responses, proliferative capacity, and the ability to generate all other subtypes of CD8^+^ T cells. The Wnt/β-catenin pathway was specifically determined as being reliant on TCF1 and LEF1 downstream signaling for mediating the expression of genes necessary for the generation of CD8^+^ T_SCM_ cells. Thus, the resultant redifferentiated iPSC-CD8^+^ T cells of Nishimura et al. may have well been one of the first cases of inducing T_SCM_ generation.

## 7. Role of TCF1 in Tregs

TCF1 may have substantial implications in determining the anti-inflammatory nature of the TME. Particularly, studies have only now begun to realize the regulatory significance of TCF1 within Tregs. A new study into the genetic regulation of Foxp3^+^ Tregs found the expression of Foxp3 to inversely correlate with levels of TCF1 and the chromatin accessibility of TCF1 targets [96]. Reducing the expression of TCF1 in Foxp3^GFPKO^ cells displayed that much of the Foxp3-induced negative modulation of chromatin accessibility may be TCF1-dependent [96]. Other studies have also reported TCF1 and LEF1 levels to be reduced in Tregs [97,98]. Nevertheless, while certain steps of the interaction between Tregs and TCF1 have been illuminated, this potential link between Foxp3 and TCF1 requires further exploration [9]. Indeed, another study published in the same year reported no such signs of reduced *Tcf7* gene expression [99].

On the other hand, studies have demonstrated a significant overlapping of the transcription factor binding sites between TCF1/LEF1 and Foxp3 [100]. Additional sharing of binding sites has been found between β-catenin and Foxp3 [101]. Given the β-catenin/TCF1 complex that is necessary for the binding of target DNA sites, inducing the expression and/or stability of TCF1 and β-catenin may deter the generation of Foxp3^+^ Tregs. This seems to be supported by the fact that the binding of β-catenin to its target binding sites adversely affects Foxp3 transcriptional regulation [101]. Such reductions in Treg levels may offer a method to modulate the suppressive TME to promote TILs.

However, the risk of certain health issues should also be brought to light when discussing the scope of utility of TCF1. Notably, recent studies have shown that the conditional deletion of TCF1 and LEF1 in Tregs resulted in spontaneous autoimmune responses [97,100]. Conversely, other studies have also shown that inducing the stable expression and functioning of the β-catenin/TCF1 complex also caused pro-inflammatory repercussions along with the absence of a suppressive regulatory mechanism [101,102]. As such, potential future therapeutic approaches depending on the modulation of TCF1 levels within Tregs ought to be done with due caution given the presence of such risks.

## 8. Conclusions: Challenges and Perspectives

Many recent studies into TCF1 and its functions have brought to light several key factors that make TCF1 a promising biomarker and/or therapeutic target. Its significance in CD8^+^ T cells for maintaining cell stemness and memory cell formation could be potentially invaluable in the treatment of cancer patients who show a diminishing population of CD8^+^ T cells able to effectively respond against and infiltrate the TME.

However, further clarity is needed in terms of how the expression of *Tcf7* and, thus, TCF1 is regulated. DNA methylation and chromatin accessibility have both been implicated by independent studies. Evidence for methylation was found in an in vitro generation of CD8^+^ T_EX_ cells using repeated antigen exposure. The suggestion of chromatin accessibility regulating *Tcf7* expression arose from T cell exhaustion studies on the effects of TOX. Confirmation of the in vitro results through in vivo studies appear to be warranted as well as a simultaneous investigation into DNA methylation, histone methylation, and histone acetylation in and around the *Tcf7* promoter.

Additionally, TCF1 appears to be multifunctional, being implicated in both exhausted and memory CD8^+^ T cells. While the effects of TCF1 on either exhausted or memory CD8^+^ T cells may have been somewhat elucidated, the relationship between TCF1^+^ T_EX_ and memory CD8^+^ T cells is less clear. Whether TCF1^+^PD-1^+^CD8^+^ T_EX_ cells are to be considered as MPECs capable of differentiating into T_CM_ cells has yet to be determined.

Nevertheless, the close association of TCF1 with inducing memory-like characteristics in CD8^+^ T cells and the strength of the anti-tumor response produced by stem-like and iPSC-CD8^+^ T cells potentially makes the presence or absence of TCF1 a valuable biomarker for determining the efficacy of ICB therapy in cancer patients. The implications of TCF1 could also justify considering TCF1 as a potential therapeutic target. Inducing the gene expression of TCF1 in antigen-specific CD8^+^ T_EX_ cells could be an alternative method of reinvigoration that bypasses the need for iPSC generation as well as a way of rescuing the memory cell forming capabilities of CD8^+^ T cells.

**Figure 2:**
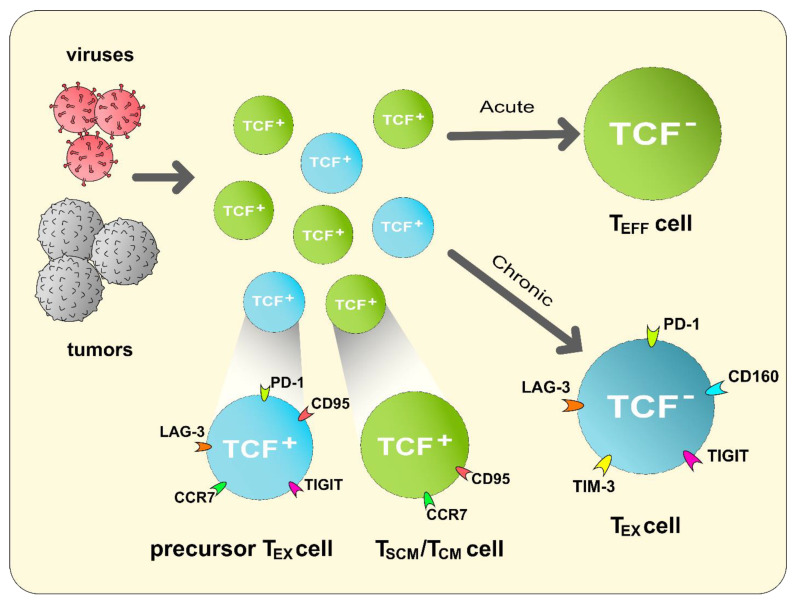
An alternative theory of CD8^+^ T_EX_ cell generation where a cell pool consisting of both precursor exhausted and non-exhausted CD8^+^ T cells is generated and expanded in response to a particular pathogenic stimulation. Depending on the acute or chronic nature of the pathogenic antigen stimulation, a particular subgroup is selected to best clear the pathogen while minimizing host cell injury.

Results showing TCF1^+^PD-1^+^ T cells as having proliferative and moderate effector capabilities may also offer alternative theories for T_EX_ cell generation. While T_EX_ cells have generally been characterized as dysfunctional, another theory has suggested that such CD8^+^ T cells are of a separate lineage of highly differentiated T cells with lessened cytotoxic effects for the protection of the host’s biology in the context of chronic infections [103]. In other words, a pathogenic challenge causes the expansion of both CD8^+^ T_EFF_ and T_EX_ cells; the acute or chronic nature of the infection may determine which subgroup of T cells is selected, respectively (Figure 2) [103]. Early research data into CD8^+^ T_EX_ cells also appear to seemingly show two distinct lineages of CD8^+^ T cells during the start of an infection: TNF-α^−^IFN-γ ^+^ and TNF-α^+^IFN-γ^+^ [104]. In fact, such data has been further clarified by the latest studies in exhaustion. Beltra et al. demonstrated, among four subsets of CD8^+^ T_EX_ cells, two subsets of progenitor TCF1^+^CD8^+^ T cells, an effector-like subset and terminally exhausted subset [105]. Moreover, additional research results seem to challenge the “dysfunctional” definition of exhaustion. A study into the characteristics of SAR-CoV-2-responding CD8^+^ T cells also found such cells to be capable of abundant IFN-γ secretion despite expressing PD-1 [12]. Furthermore, another recent study demonstrated that markers of exhaustion, including ID3, PD-1, and TOX, could be observed since the TCF1^+^ precursor stage of CD8^+^ T cells [106]. Provided that TCF1^+^ cells have clearly been shown to possess potent effector functions, there seem to be indications for a need to reconsider current models of T cell exhaustion. Additionally, approaching CD8^+^ T cell exhaustion from an alternate view may identify TCF1^+^ T_EX_ cells as a highly proliferative cell population necessary in the maintenance of “exhausted” T_EFF_ cell populations for a slow and controlled immune response against prolonged infections and/or human diseases [107].

Finally, the significance may extend beyond ICB immunotherapy to also affect chimeric antigen receptor (CAR)-T cell-based therapies. A study exhibiting the potency of IL-9-secreting (T9) CAR-T cells in tumor control demonstrated that such CAR-T cells possess a T_CM_ cell phenotype, are highly proliferative, and capable of generating T_EFF_ cells [108]. Given the essential nature of TCF1 in generating memory T cells, especially of the T_CM_ subtype, and the proliferative nature of TCF1^+^ CD8^+^ T cells and their ability to differentiate into SLECs, it may be plausible to assume that one of the main regulating transcription factors in these T9 CAR-T cells may be TCF1. Unfortunately, the scope of this study was not inclusive of TCF1 and further studies should be conducted to confirm the presence and activity of TCF1. Additionally, these T9 CAR-T cells were found to undergo reduced activation-induced cell death, potentially due to their downregulation of Fas and Fas ligands [108]. This may also suggest a possible mechanism by which TCF1^+^ CD8^+^ T cells in general exhibit a higher survivability than other CD8^+^ subgroups.

Although much about CD8^+^ T_EX_ cells and methods for their reinvigoration still needs to be uncovered, research has been hampered by the limited number of CD8^+^ cells able to be isolated for the study. Until now, most research into T cell exhaustion has relied on in vivo experiments. However, in vivo methods of inducing and isolating CD8^+^ T_EX_ cells produces a low yield and are relatively time-consuming. Until recently, attempts at creating in vitro methodologies for creating T_EX_ cells were not successful in producing *bona fide* exhausted cells [109]. However, a recent study into in vitro methods of T_EX_ cell generation shows great promise for potential use. Using repeated antigen exposure in conjunction with IL-7 and IL-15 treatment, CD8^+^ T_EX_ cells possessing characteristics similar to that of CD8^+^ T_EX_ cells found in the LCMV exhaustion models were able to be generated [19]. While the nature of CD8^+^ T cell exhaustion varies between types of infections and human disease as well as amongst different types of tissue, such development of in vitro methods for exhaust T cell generation may be utilized as a means of accelerating an otherwise slow and inefficient field of T cell research [110].

TCF1 represents a new potential direction for research into CD8^+^ T_EX_ cells as well as Tregs, and research groups studying TCF1 and its effects have started to increase rapidly in very recent years as noted by the number of studies published on TCF1. Through the use of such new methodologies, studies into the effects of inducing TCF1 expression in CD8^+^ T_EX_ cells may be accelerated and may even lead to new findings with implications that tie to research in the fields of stem cells and developmental biology, given the relationship of TCF1 with stemness-related gene expression.

## Figures and Tables

**Table 1:** Cell surface markers of the different memory CD8^+^ T cell subsets [52,53,54,55,56].

	Naïve	T_SCM_	T_CM_	T_EM_
*CD45RA*	+	+	−	−
*CD45RO*	−	+	+	+
*CCR7*	+	+	+	−
*CD62L*	+	+	+	−
*CD27 (TNFR)*	+	+	+	−
*CD28*	+	+	+	+/−
*CD95 (Fas)*	−	+	+	+
*CD122 (IL-15Rβ)*	−	+	+	+
*CD127 (IL-7Rα)*	−	+	+	−

## Data Availability

Not applicable.

## Abbreviations

APC	antigen-presenting cell
CAR	chimeric antigen receptor
CTL	cytotoxic T lymphocytes
CTLA-4	cytotoxic-T-lymphocyte-associated protein 4
DC	dendritic cell
FDA	(U.S.) Food and Drug Administration
ICB	immune checkpoint blockade therapy
IFN	interferon
iPSC	induced pluripotent stem cell
MHC	major histocompatibility complex
MPEC	memory precursor effector cell
LEF	lymphocyte enhancer factor
MDSC	myeloid-derived suppressor cell
PD-1	programmed cell death 1
PD-L	programmed cell death ligand
SLEC	short-lived effector cell
T9 CAR-T	interleukin-9-secreting chimeric antigen receptor-T cell
TAM	tumor-associated macrophage
TCF1	T cell factor 1
T_CM_	central memory T cell
TCR	T cell receptor
T_EFF_	effector T cell
T_EM_	effector memory T cell
T_EX_	exhausted T cell
TIGIT	T cell immunoreceptor with Ig and ITIM domain
TIL	tumor-infiltrating lymphocyte
TME	tumor microenvironment
Treg	regulatory T cell
T_RM_	tissue-resident memory T cell
T_SCM_	stem-cell memory T cell
WRE	Wnt-response element
